# Revision of *Polygonatum* (Asparagaceae, Nolinoideae, Polygonateae) of Taiwan

**DOI:** 10.3897/phytokeys.117.31902

**Published:** 2019-02-14

**Authors:** Chao Chien-Ti, Tseng Yen-Hsueh

**Affiliations:** 1 School of Life Science, National Taiwan Normal University, No. 88, Tingchou Rd. 4 section, Wenshan district, Taipei city 116, Taiwan National Taiwan Normal University Taipei Taiwan; 2 Department of Forestry, National Chung Hsing University, No. 145, Xinda Rd., South district, Taichung city 402, Taiwan National Chung Hsing University Taichung Taiwan

**Keywords:** new combination, *
Polygonatum
*, plant taxonomy, Taiwan

## Abstract

*Polygonatum* is widely distributed in the northern hemisphere, especially in eastern Asia. There has been no comprehensive taxonomic study of Taiwanese taxa for some time, and researchers could not agree on a consistent treatment of the genus. Therefore, we revised the genus by literature review and type specimen examination along with comparison of morphology, karyotype and pollen characteristics. Only one species *P.arisanense* Hayata was recognized in this study. *P.chingshuishanianum* Ying and *P.formosanum* (Hayata) Masamune & Simada are regarded as varieties of *P.arisanense* and are here presented as two new combinations, P.arisanensevar.chingshuishanianum and P.arisanensevar.formosanum.

## Introduction

The genus *Polygonatum* comprises ca. 60 species, distributed in the northern hemisphere, mainly from southwest China to Japan (Chen et al. 2000). Some species, such as *P.sibiricum* Redoute and *P.odoratum* (Mill.) Druce have been cultivated as medical or horticultural crops (Wujisguleng et al. 2012, Tang et al. 2018).

The diagnostic characters of the genus include the presence of rhizomes, single and often arching stem, alternate, opposite, or whorled phyllotaxis, axillary inflorescences with one to several flowers, perianth tubes longer than segments, and berries (Wang et al. 1978; Chen et al 2000; Ying 2000). The basic chromosome number was x=9–11 (sect. Polygonatum), 12 (sect. Sibirica), 13–15 (sect. Verticillata)(Floden and Schilling 2018).

The taxonomy of *Polygonatum* on Taiwan has varied significantly depending on the authors since their earliest collections. The first collection of *Polygonatum* in Taiwan was made by Matsumura and Hayata (1906) who reported it as P.officinaleAll.var.maximowiczii Franch. & Sav. This record was followed by Hayata (1908) and Kawakami (1910). Later, Hayata (1915) described a new species *P.alte-lobatum* Hayata. These taxa were listed in the plant list of Hayata (1917). Later, Hayata (1920) described two new taxa *P.arisanense* Hayata and P.officinaleAll.var.formosanum Hayata, and the earlier record of P.officinalevar.maximowiczii was a misidentification of P.officinalevar.formosanum. Sasaki (1928) followed the example of Hayata (1920) but applied a different name P.japonicaC. Morren & Decne.var.formosanum Hayata. Masamune and Simada (1936) upgraded P.officinalevar.formosanum to species level as *P.formosanum* (Hayata) and this was followed by Masamune (1954). Ying (1969) treated *P.arisanense* and *P.formosanum* as synonyms of *P.cyrtonema* Hua, and described a new species *P.daitonense* Ying in his master’s thesis, but without formal publication afterwards. Liu and Ying (1978) listed *P.alte-lobatum* and *P.cyrtonema* in the Flora of Taiwan, 1^st^ edition. Wang et al. (1978) listed three Taiwanese species of *Polygonatum*: *P.alte-lobatum*, *P.arisanense*, and *P.odoratum* (Mill.) Druce. This treatment was followed by Chen et al. (2000). Jeffery (1980) treated *P.arisanense* as a synonym of *P.cyrtonema*. Ying (1988) described a new species *P.chingshuishanianum* Ying from eastern Taiwan. Ying (1990) revised the genus and catalogued three taxa in Taiwan: *P.alte-lobatum*, *P.chingshuishanianum*, and P.odoratum(Mill.)Drucevar.pluriflorum (Miq.) Ohwi. This treatment was also followed by Ying (2000) and Boufford et al. (2003). Wang (1997) studied the karyotype of Polygonateae of Taiwan and recorded two species, *P.altelobatum* and *P.cyrtonema*. The aforementioned *P.chingshuishanianum* was treated as a synonym of *P.altelobatum*. Based on morphological data, Chao et al. (2013) transferred *P.alte-lobatum* to *Heteropolygonatum* M. N. Tamura and Ogisu as a new combination: *H.alte-lobatum* (Hayata) Y. H. Tseng, H. Y. Tzeng and C. T. Chao.

From the history outlined above, it is clear that almost every author proposed their own treatment, and no comprehensive revision has been published. Some taxa, like *P.chingshuishanianum*, were poorly known; very few specimens could be examined in herbaria, and the available specimens, including the type specimen, all lacked floral parts. Therefore, it was deemed that a modern review of *Polygonatum* of Taiwan was necessary. The aims of this study were to elucidate the taxonomic status and circumscription of each taxon, together with their nomenclature and distribution in Taiwan.

## Methods

### Study materials

The study materials were from herbaria and field collections with at least 3 individuals cultivated for each population. Living materials were cultivated in the greenhouse of the Department of Forestry, National Chung Hsing University. Voucher specimens (include living ones) were deposited in the herbarium of the National Chung Hsing University (TCF). The following herbaria were examined: CHIA, HAST, KYO, NTUF, PPI, TAI, TAIE, TAIF, TCF, TI, TNM, and TNU. We also retrieved type specimen images of *Polygonatum* from the website of the Muséum National d’Histoire Naturelle (P). Pollen observation and karyotype analysis materials are listed in Table 1.

**Table 1.** Materials of pollen and chromosome observation in this study.

Taxa	Location	Coll. no.
Polygonatum arisanense var. arisanense	Nantou county: Hsinyi township, Mt. Chungtashan	Chao 4131
	Pingtung county: Wutai township	Chao 4019
P. arisanense var. chingshuishanianum	Hualien county: Hsiulin township, Mt. Chingshuishan	Chao 4128
P. arisanense var. formosanum	Taipei city: Yangmingshan national park, Mt. Tatun main peak	Chao 4105

The floral morphology is important in the taxonomy of *Polygonatum*, and therefore we had to check the flower morphology of living plants, especially *P.chingshuishanianum*, whose floral part was lacking on the type specimen. In order to do this, we visited the type population of *P.chingshuishanianum* for several years, and were only able to capture two anthesis individuals.

### Pollen morphology

The pretreatment of the anther followed the method of Halbritter (1998). The anther was dried with a critical point dryer (Quoram E3100). The pollen was taped on to the stub with copper tape, after sputter-coating with gold (Quoram SC7620) and observed by SEM (Hitachi S-3400N). The terminology and description of pollen morphology were in accordance with Punt et al. (2007) and Hesse et al. (2009) while the aperture type classification followed the procedure of Halbritter and Hesse (1993).

### Karyotype analysis

Plant materials for karyotype analysis were cultivated in the greenhouse of the Department of Forestry. Root tips were collected in the morning on a sunny day and preserved in 0.002M 8-hydroxyquinoline solution below 10 °C for eight hours. Afterwards, the roots were fixed in Carnoy’s solution (glacial acetic acid: 99.5% EtOH, 1:3) at 4 °C overnight. The fixed roots were stained with acetic-orcein overnight, squashed and observed under a light microscope (Accu-Scope 3025 Series). Cells showing good chromosome spreading were photographed with a CCD camera (ProgRes C14 Plus). Karyotype analysis was done according to the procedures of Levan et al. (1964) and Stebbins (1971).

### Distribution and conservation rank evaluation

The distribution maps were made by the location of herbaria specimens and our field work. Older specimens were geo-referenced using the study of Huang et al. (1993), and points proximal to written locality were mapped. Classification of geographical climatic regions and altitudinal vegetation zones followed the guidelines of Su (1984, 1985). We used the protocol of the red list of vascular plants of Taiwan, 2017 (editorial committee of red list of vascular plants of Taiwan, 2017) for evaluation of conservation ranks.

## Results

### Diagnostic characteristics of *Polygonatum*

Leaf

Leaf morphology is an important character for identification of intrageneric taxa of *Polygonatum* (Chen et al. 2000). The leaf shape of P.arisanensevar.chingshuishanianum is lanceolate to oblong lanceolate with obtuse apex (Fig. 7C, D), whereas the leaves of P.arisanensevar.arisanense and P.arisanensevar.formosanum are ovate to ovate-lanceolate with acute and obtuse apexes respectively (Fig. 6C, D, 8C, D). The texture also differs among these taxa. The leaf of P.arisanensevar.arisanense is chartaceous, whereas P.arisanensevar.chingshuishanianum and P.arisanensevar.formosanum have chartaceous to coriaceous leaves.

Rhizome

The Taiwanese taxa all have tuberous rhizomes that are similar in appearance to one another, rather than the terete rhizomes found in *P.odoratum* (Fig. 6B, 7B, 8B).

Inflorescence

The inflorescences of *Polygonatum* species are solitary to multi-flowered umbels, axillary and often pendulous. The three taxa of Taiwan have similar inflorescence forms, but can be distinguished by the number of flowers within an inflorescence. The inflorescences of P.arisanensevar.chingshuishanianum have only one or two flowers, while P.arisanensevar.arisanense and P.arisanensevar.formosanum have (3–)5–7 and 2–3(–5) flowers, respectively (Fig. 6E, 7E, 8E).

Flower

The flowers of the three taxa are typical for *Polygonatum*. The perianth tube of P.arisanensevar.chingshuishanianum is smaller (6–8 mm × 4–5 mm) than the others. Besides, two forms of perianth tube are found among the taxa of Taiwan. The first one is found in P.arisanensevar.arisanense, which have an acute base (less than 90°)(Fig. 6F). The second type is found in P.arisanensevar.chingshuishanianum and P.arisanensevar.formosanum, which have an obtuse to truncate base (more than 90°)(Fig. 7E, 8F).

Pollen morphology

**Figure 1.**
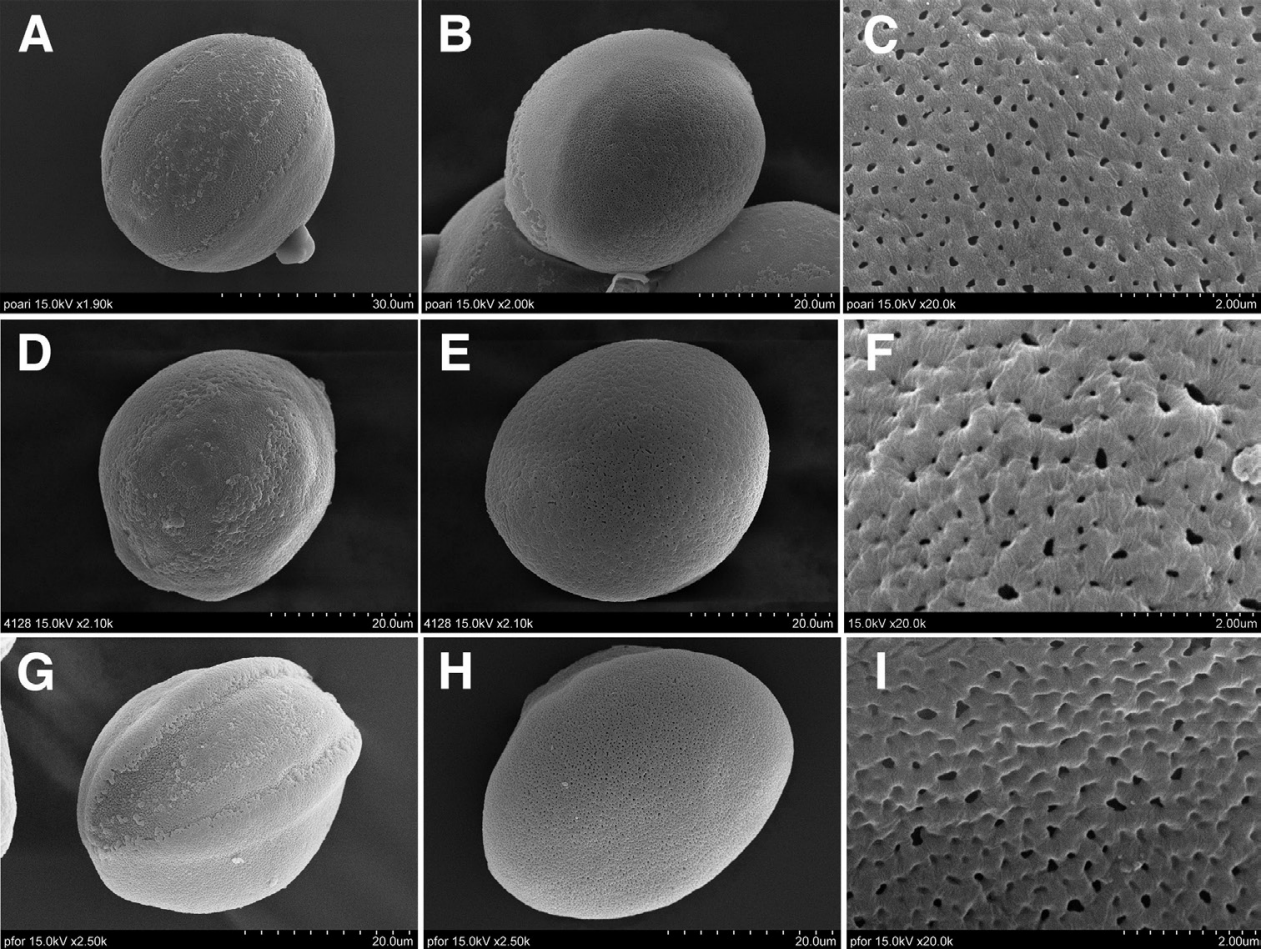
Pollen morphology of *Polygonatum* of Taiwan. **A–C**P.arisanensevar.arisanense**D–E**P.arisanensevar.chingshuishanianum**G–I**P.arisanensevar.formosanum**A, D, G** proximal polar view **B, E, H** distal polar view **C, F, I** sculpture.

The pollen grains of Taiwanese *Polygonatum* are monosulcate monads of medium size, spheroidal, simple-sulcate aperture type, and the proximal polar view is perforate. The widths of the lumina and the muri are similar among the three taxa. No conspicuous difference is found among them (Table 2, Fig. 1).

**Table 2.** Pollen morphology of *Polygonatum* taxa of Taiwan.

**Taxa**	**Equatorial axis (μm)**	**Polar axis (μm)**	**P/E**	**Shape**	**Size rank**	**Aperture**	**Sculpture**
*P.arisanense* var. arisanense	28.03±0.66	24.07±0.83	0.86	spheroidal	medium	simple-sulcate	perforate
P. arisanense var. chingshuishanianum	38.03±3.03	32.81±3.37	0.86	spheroidal	medium	simple-sulcate	perforate
P. arisanense var. formosanum	40.87±0.26	31.34±2.00	0.77	spheroidal	medium	simple-sulcate	perforate

Karyotype analysis

**Figure 2.**
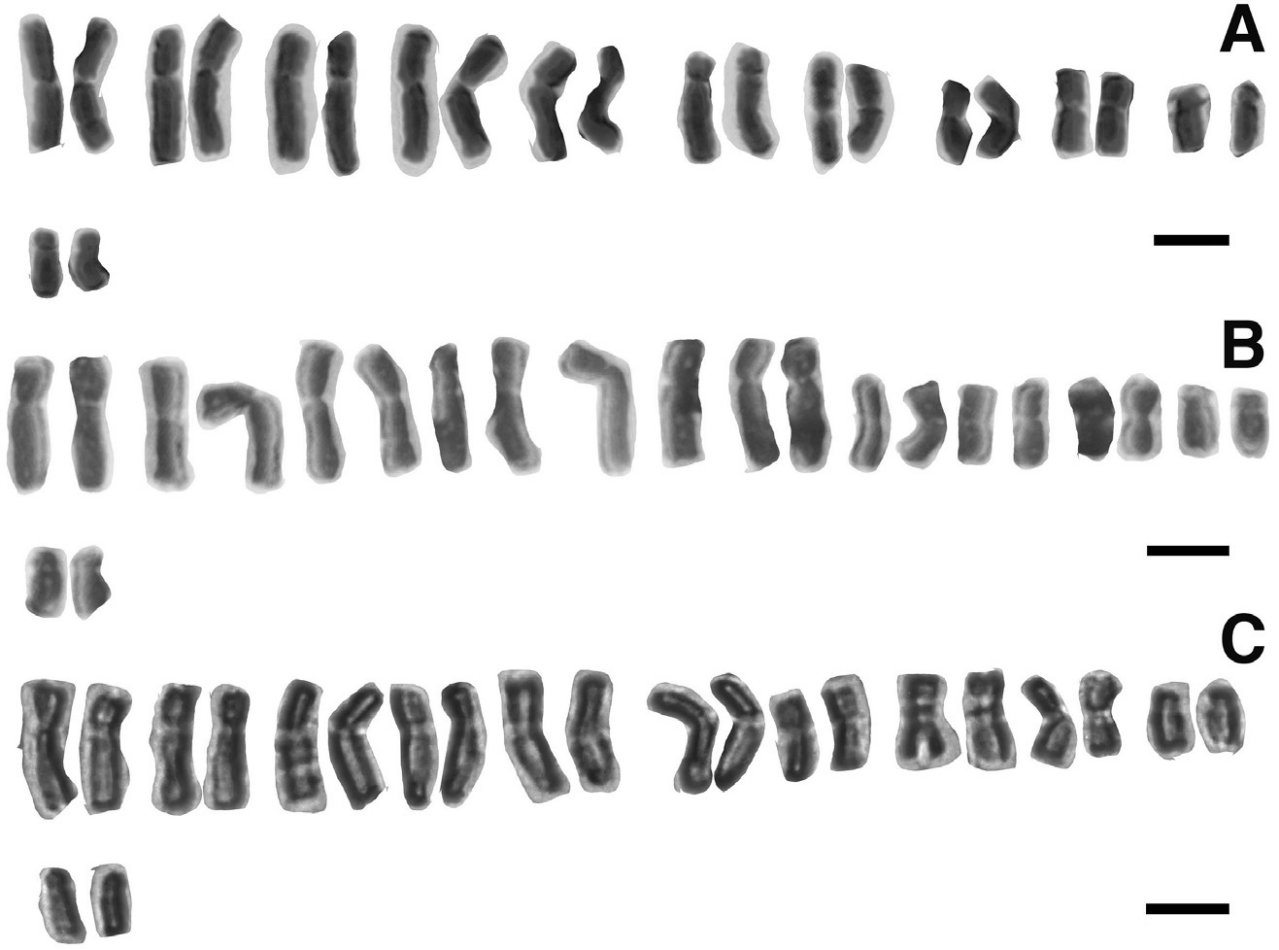
Chromosome of *Polygonatum* of Taiwan. **A**P.arisanensevar.arisanense**B**P.arisanensevar.chingshuishanianum**C**P.arisanensevar.formosanum. Scale bar: 5 μm.

The chromosome number of the three taxa is determined to be 2n = 2x = 22, and the asymmetry type is 2B. The chromosomes exhibited centromeres at the median (m), submedian (sm), and terminal (t) positions. The numbers of each type were slightly different among the varieties, and no secondary constriction was observed. The karyotype formula of P.arisanensevar.arisanense is 2n = 22 = 10m+9sm+3^st^, P.arisanensevar.chingshuishanianum is 2n = 22 = 9m+12sm+2^st^, and P.arisanensevar.formosanum is 2n = 22 = 10m+10sm+2^st^ (Table 3, Fig. 2).

**Table 3.** Karyotype of *Polygonatum* of Taiwan.

Taxa	Chromosome number	Karyotype formula	Asymmetry type
P. arisanense var. arisanense	22	10m+9sm+3^st^	2B
P. arisanense var. chingshuishanianum	22	9m+12sm+2^st^	2B
P. arisanense var. formosanum	22	10m+10sm+2^st^	2B

Distribution

**Figure 3.**
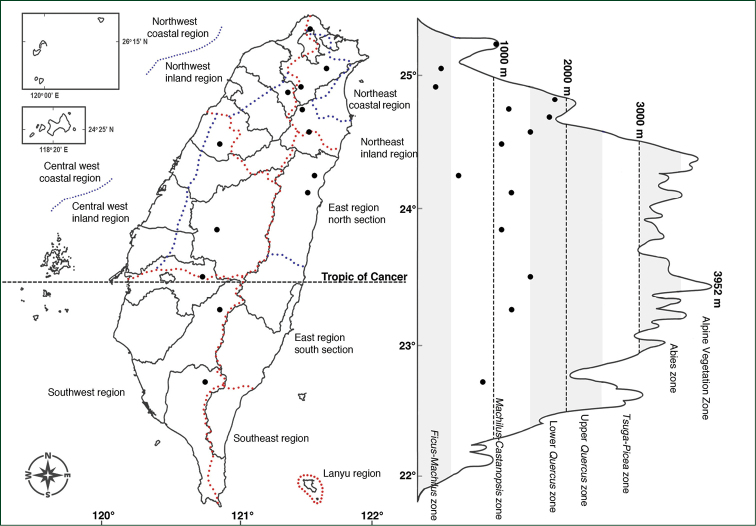
Distribution map of Polygonatumarisanensevar.arisanense.

The taxa of *Polygonatum* of Taiwan are all endemic, and were found from low to medium altitude in mountainous regions. Compared to other varieties, P.arisanensevar.arisanense has the widest distribution. It grows in most geographical climate zones except for the Lanyu region, the Southeast, the south section of the East region, and other coastal areas. The vertical distribution of this variety is from ca. 300 m up to 2000 m, lower in the northern part of Taiwan. This region is located in the *Ficus*-*Machilus* zone to lower *Quercus* zone of the altitudinal vegetation zone classified by Su (1984), which had more precipitation and higher relative humidity (Fig. 3). The habitats are often shady with high moisture, under, or in the margin, of a forest.

**Figure 4.**
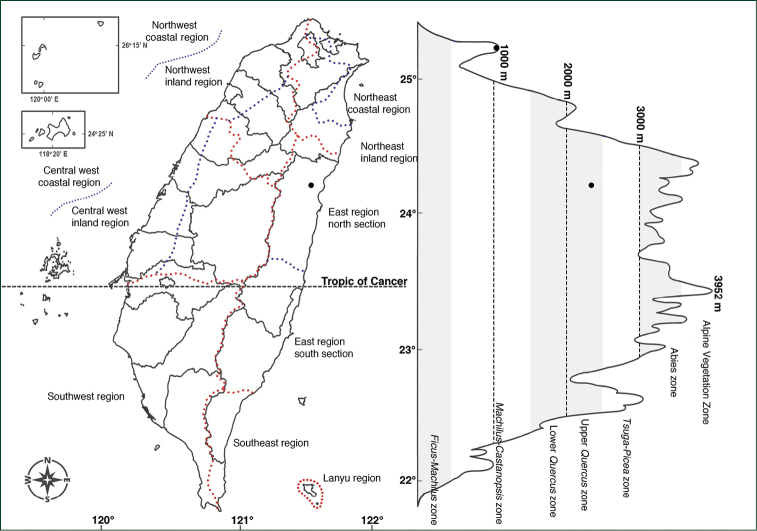
Distribution map of Polygonatumarisanensevar.chingshuishanianum.

**Figure 5.**
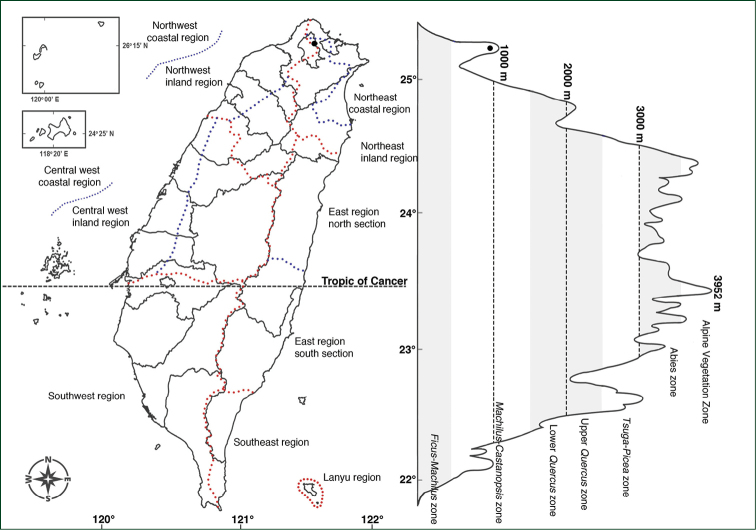
Distribution map of Polygonatumarisanensevar.formosanum.

In contrast, P.arisanensevar.chingshuishanianum and P.arisanensevar.formosanum have very narrow distributions, especially the former. P.arisanensevar.chingshuishanianum is only found in one location, near the summit of Mt. Chingshuishan in Hualien county of eastern Taiwan (Fig. 4). The plants are growing on a limestone slope with full sunlight and thin soil. On the other hand, P.arisanensevar.formosanum is only found in the region of Yangmingshan National Park, a volcanic area in northern Taiwan (Fig. 5). The populations were found from the exposed roadside to the forest habitat.

Conservation rank

In the red list of vascular plants of Taiwan 2017 (editorial committee of the red list of vascular plants of Taiwan, 2017), the conservative rank of *P.chingshuishanianum* (=P.arisanensevar.chingshuishanianum) and P.odoratumvar.pluriflorum (=P.arisanensevar.arisanense and P.arisanensevar.formosanum) was evaluated as data deficient (DD) and of least concern (LC). Here we reevaluate three taxa of *Polygonatum* of Taiwan. As a result, P.arisanensevar.arisanense is evaluated at LC rank. The population was still large in the island and the plants are common, so there is no immediate threat to this taxon. Polygonatumarisanensevar.chingshuishanianum is ranked as critical endangered (CE)(A1(a), B1(bii+biii), D1), because there are few fertile individuals and there is a hiking trail that crosses the population area. Polygonatumarisanensevar.formosanum is evaluated as near threatened (NT)(C, C1). This variety is only found in Yangmingshan National Park, but there are more than ca. 10,000 fertile individuals and they are regenerating well. However, considering the narrow distribution and the fact that some of the populations are near places of human activity, we evaluate the conservative rank as near threatened.

## Discussion

Here we present evidence that leads us to conclude that there is but one species and three taxa of *Polygonatum* of Taiwan. These are enumerated below.

### The differences between *Polygonatum*arisanense and related taxa

*Polygonatumarisanense* were identified as *P.odoratum*, P.odoratumvar.pluriflorum or *P.cyrtonema* by several authors (Ying 1969, 1990, 2000; Wang et al. 1978; Jeffery 1980; Wang 1997; Boufford et al. 2003). These species all have similar appearances with alternate leaves, pendulous axillary inflorescences, white perianth tubes, and purplish berries. Identification of species often relies on the rhizome and stamen morphology (Tamura 1993; Chen et al. 2000), but such parts are often neglected when collecting the samples, or are hard to observe in herbarium sheets. The morphology of the rhizome provides significant value in determining the taxonomy of *Polygonatum*, but this character is usually lacking on the herbarium specimens, even on the type specimens. Different forms can be recognized among the taxa; for example, *P.hookeri* Baker has terete rhizomes, while *P.franchetii* Hua and *P.filipes* Merr. *ex* C. Jeffery & McEwan have moniliform ones. Tuberous rhizomes are found in all Taiwanese taxa, and are different from the terete rhizomes of *P.odoratum*. Therefore, misidentification is relatively common in *Polygonatum* species. According to literature review, and examination of living and herbarium materials, *P.arisanense* is distinguished from *P.odoratum* by having tuberous rhizome, which is terete in the latter. On the other hand, *P.arisanense* was distinguished from *P.cyrtonema* by its filaments being adnate to the middle of the perianth tube, and the anther being without spurs, whereas the filaments are adnate near the apex of the perianth, and the anthers are spurred in the latter. Such a result was also supported by the molecular study (Floden and Schilling 2018) The fact that the chromosome number of *P.odoratum* was 2n = 20 (Tamura 1990), and of *P.arisanense* was 2n = 22, revealed that the difference between them was not only on the morphological, but also on the genetic level (Table 4). Besides, Hsu (1971) also recorded a tetraploid individual (2n = 44) of *P.arisanense* from Taitung county, implying that the polyploidy events may be found in some populations. Such events were often found in the populations of *Polygonatum* (Therman 1950; Tamura 1990). Further study of the distribution and frequency of polyploidy event of *P.arisanense* population is needed.

**Table 4.** Comparison of *Polygonatumarisanense* and related species.

	* P. arisanense *	* P. cyrtonema *	* P. odoratum *
Rhizome	tuberous	moniliform or tuberous moliniform	terete
Stamen	not spurred	spurred	not spurred
Chromosome number	2n=22	2n=18, 20, 22	2n=20

### The taxonomic status of *P.chingshuishanianum* and *P.formosanum*

In order to determine the taxonomic status of *P.chingshuishanianum*, the floral morphology is the key character due to the lack of type specimen. The observed flower morphology was generally identical to that of Ying (1988, 2000), and the morphology of other parts matched those of the type specimen. Therefore, the plant we found was identified as *P.chingshuishanianum*, and was the first record of floral morphology after the original publication of Ying (1988). Generally, the floral morphology was similar to *P.arisanense*, but minor distinctions included the fact that there were few-flowered inflorescences, smaller flowers, truncate to obtuse perianth tube base, dwarf plant size and thicker leaves. These were all different from typical *P.arisanense*. The morphology of the pollen and karyotype were also similar to *P.arisanense*, but the floral morphology was distinct from the lower altitude population of P.arisanensevar.arisanense. Moreover, the habitat of this taxon was on the limestone region of eastern Taiwan, which had abundant endemic species found in this region, such as *Dianthusseisuimontanus* Masamune (Caryophyllaceae), *Berberischingshuiensis* T. Shimizu (Berberidaceae), and *Rhamnuschingshuiensis* T. shimizu (Rhamnaceae). Such a unique geological environment may lead to unique evolution events. Therefore, we treated this taxon as a variety of *P.arisanense* rather than an independent species as Ying (1988, 2000)(Table 5).

**Table 5.** Comparison between *Polygonatumarisanense* and its variaties.

	var. arisanense	var. chingshuishanianum	var. formosanum
Leaves shape	lanceolate to wide lanceolate	lanceolate to oblong lanceolate	ovate to ovate lanceolate
Texture	chartaceous	chartaceous to thinly coriaceous	thick chartaceous to coriaceous
Flowers per inflorescence	5–7	1 or 2	3–5
Flowers perianth tube size (cm)	1.5–2 × 1	1–1.5 × 0.8	2–3 × 0.5–1
Base of perianth tube	acute	flattened	flattened

Polygonatumofficinalevar.formosanum was originally described by Hayata (1920). and elevated to *P.formosanum*Masamune and Simada (1936). From nearly that time henceforth it has been treated as a synonym of *P.odoratum*, P.odoratumvar.pluriflorum, or as *P.cyrtonema* (Liu and Ying 1978; Wang 1997; Chen et al. 2000; Ying 2000). After careful observation of the types and living plants from the type locality, this taxon was found to be more similar to *P.arisanense* rather than the species and taxa mentioned above. This taxon differs from var.arisanense by having thicker leaves, larger flowers, basal flattened perianth tubes, and fewer-flowered inflorescences, but the pollen morphology and karyotype are similar to those of *P.arisanense*, so we prefer to treat this taxon as a variety of *P.arisanense* (Table 5).

## Taxonomic treatment

### Key to *Polygonatumarisanense* and its varieties

**Table:** 

1	Leaves lanceolate to ovate; flowers usually 3–7 per inflorescence	**2**
–	Leaves lanceolate to oblong-lanceolate; flower solitary or 2 per inflorescence	** P. arisanense var. chingshuishanianum **
2	Leaves lanceolate to wide lanceolate, chartaceous; perianth tube base slightly acute	** P. arisanense var. arisanense **
–	Leaves ovate to lanceolate-ovate, thick chartaceous to coriaceous; perianth tube base flattened	** P. arisanense var. formosanum **

#### 1. Polygonatum arisanense Hayata var. arisanense

Taxon classificationPlantaeAsparagalesAsparagaceae

, Icones Plantarum Formosanarum. 9:140. 1920. Type: Taiwan, Chiayi County, Arisan, Kodensho, leg. S. Sasaki, May 1913. (Lectotype designated by Jeffery (1980): TI!; isotype: TI!)

Fig. 6 萎蕤

P. cyrtonema sensu auct. Liu & Ying non. Hua in Journal de Botanique (Morot) 6 (21): 393–394. 1892.
P. odoratum sensu auct. Chen et al. non. Druce in Annals of Scottish Natural History 60: 226. 1906.
P. odoratum (Mill.) Druce var. pluriflorum sensu auct. Ying non. Ohwi in Bulletin of the Natural Science Museum 26: 7. 1949.

##### Perennial herbs.

Rhizome tuberous. Stem arching, 30–150(–200) cm long, green or purplish green, glabrous, sometimes covered with white powder, base covered with scale leaf, caducous. Leaf deciduous, alternate, chartaceous, 3-multiple nerved, lanceolate to wide lanceolate, 15.0–18.5 cm long, 5.5–8.0 cm wide, apex acuminate, base attenuate, petiole short, 3–5 mm long, often reddish or purplish. Inflorescences umbel with 5–7 flowers, peduncle longer than pedicels, ca. 3 cm long, glabrous, inflated, articulated close to flowers, bracteoles very minutely, caducous. Flowers pendulous, perianth tube 1.5–2.3 cm long, ca. 1 cm in diam., white with pale green veins, base acute, perianth segments 6, arranged into 2 whorls, each 3, 5–10 mm long, ca. 5 mm wide, green with dark green strip, ovate to triangle, apex obtuse, floccose. Stamen 6, filaments slender, base expansion and flattened, papillose or short cottony, inserted at middle of perianth tube, ca. 1 cm long, anthers ca. 3 mm long, 2-loculed, introrse and longitudinally dehiscent. Ovary superior, 3-loculed, ovate to oblong, covered with white powder, 3.0–4.5 mm long, 2.0–3.5 mm in diam., apex obtuse; style filiform, 1.3–1.5 cm long, stigma entire, pubescent. Fruits berry, purplish black. Seeds numerous. 2n=22.

##### Endemic.

Distributed from low to ca. 2000 m mountains through the island (Fig. 3).

##### Specimen examined.

**Taipei city**: Nankangshan, 30 m, 20 May 1987, S. M. Chaw 419 (HAST); **New Taipei city**: Wuliaochien, 500 m, 20 Aug 2004, C. M. Wang et al. 7627 (TNM); In silvis inter Sauken et Hyarawa, T. Suzuki 7047 (TAI); tonroku ridge of kahoyama, 11 May 1935, Fukuyama ST19237 (TAI); rarayama, 1200 m, 7 Sep. 1934, T. Suzuki 11733 (TAI); Lo-pei-shan, Y. B. Cheng & T. S. Hsieh 1182 (TAI); Mt. Wuliaochien, 300–500 m, 26 Apr 2014, T. C. Hsu 6959 (TAIF); Mt. Peichatienshan, 1500 m, 10 Oct 1984, C. I Peng 7466 (HAST); Tachienchih, 700 m, 24 Mar 2001, Y. Y. Huang 280 (HAST); Shihting, 400 m, 28 Feb 1993, H. W. Lin et al. 106 (TNU); **Ilan county**: Chiaping logging trail, 1000–2000 m, 7 Aug 2006, C. H. Chen et al. 7442 (TNM); ca. 8 km of no. 100 logging trail of Chilan, ca. 1300 m, 27 Apr 2007, C. F. Chen et al. 2501 (TNM); Tatung, T. Y. A. Yang et al. 13664 (TNM); Mt. Taiheizan, Jul. 1929, S. Suzuki 1072 (TAI); Mt. Taiping, 27 Aug. 1962, Chuang et al. 4795 (TAI); Shi-yen-ya-ko, C. S. Kuoh 1789 (TAI); Mt. Taiping, C. C. Chuang et al. 4689 (TAI); Mingchih, Liao et al. 10531 (CHIA); Taipingshan logging trail, 29 Apr 2007, P. F. Lu 13696 (TAIF); Ssuchi logging trail, 1400–1700 m, 9 May 2011, T. C. Hsu 3977 (TAIF); Mt. Poluo, 1300 m, 17 Apr 2014, C. T. Chao 3375 (TCF); near Chilan, on provincial road sign between 67 km to 69 km, 1200 m, 14 Aug. 1997, J. C. Wang & C. H. Chen 10525 (TNU); 710 logging track, 1000–1100 m, 22 Sept 1996, Y. C. Chen 65 (TNU); **Taoyuan county**: Mt. Lala, 1500 m, 7 May 1986, S. Y. Lu 19144 (TAIF); same loc., ca. 2 Jul 2009, C. T. Chao 785, 786, 787 (TCF); same loc., 26 Sep 2009, C. T. Chao 1033, 1034, 1035, 1036 (TCF); on the way from Lalashan forest recreation area to Fushan, 1500–1600 m, 11 May 1997, S. M. Kuo 82 (HAST); Pafu ancient trail, 1500 m, 14 Apr 2014, C. T. Chao (TCF); from Lalashan forest recreation area to Fushan, 1500–1600 m, 11 May 1997, S. M. Kuo et al. 82 (TNU); **Hsinchu county**: Yuanyanghu, 1700 m, 9 May 1995, H. Y. Shen et al. 778 (TNM); same loc., Y. F. Wang 1043 (TAI); same loc., 1700 m, 28 Jun 1985, S. Y. Lu 16589 (TAIF); same loc., 1670 m, 8 May 1995, H. Y. Shen 746 (HAST); Malo to Shangyulao, ca. 1000 m, 14 May 2004, C. M. Wang & C. P. Lu 7371 (TNM); Taikeigun, Tamankei no minamoto, T. Suzuki & T. Nakamura ST 18228 (TAI); Chenhsipao, ca. 1800 m, 26 Jul 2000, Summer Collecting Team 11595 (TAIF); same loc., 1640 m, 10 May 2000, C. H. Lin 289 (HAST); Mt. Litung, ca. 1800 m, 17 May 2008, P. F. Lu 15952 (TAIF); same loc., 1510 m, 3 Jul 2002, C. H. Chen 4374 (TAIE); Ssumakussu, 1500 m, 23 May 2012, T. C. Hsu 5757 (TAIF); Taohsia to Shangyulao, 1300 m, 2 Jun 2012, T. C. Hsu 5789 (TAIF); Kuanwu, Talu forest road 20–26 km, 2000 m, 20 May 1994, J. C. Wang 9214 (HAST); the north line of Loshan logging trail, 1440 m, 9 Jun 2004, C. C. Wu 677 (HAST); Chienshih, ca. 1000 m, 20 Jan 2010, C. T. Chao 1271 (TCF); Tulungtan, 2000 m, 17 Apr 2016, C. T. Chao 4095 (TCF); Matai ancient trail, 500 m, 7 Dec 2014, C. T. Chao 3622 (TCF); Kuanwu, Talu forest road 20–26 km, 2000 m, 20 May 1994, J. C. Wang et al. 9214 (TNU); **Miaoli county**: Talu logging trail, C. H. Yu 950 (TAI); Mt. Henglung ancient trail, ca. 1550 m, 25 Apr 2010, P. F. Lu 20163 (TAIF); Kuantaoshan, 700 m, C. T. Chao 1287 (TCF); Hennungshan, 1000 m, 8 Jan 1997, M. Y. Shen 1277, 1278 (TAIE); Hoununghsi, 5 Jun 1997, M. Y. Shen 1727 (TAIE); Malabanshan, 1402 m, 22 Mar 2006, M. Y. Shen 4318 (TAIE); Ta-lu logging tract east line, 2000 m, 8 Jul 1998, J. C. Wang & summer collection team 10662 (TNU); **Nantou county**: Hakku-Musha, 14 Jul. 1930, G. Masamune 1349 (TAI); Shanlinhsi, K. C. Chang 137 (CHIA); Chitou, 15 Feb 1960, T. I. Chuang 3167 (HAST); same loc., 1150 m, 5 Feb 2010, C. T. Chao 1326 (TCF); Renlun logging track, 1638 m, 27 Jun 2017, M. Y. Shen 5393 (TAIE); Tungpu to kuankao, 1700 m, 14 Apr 1996, K. C. Yang 4692 (TNU); Hsitou recreation area, 1200–1500 m, 14 Feb 1997, S. M. Kuo et al. 71 (TNU); **Chiayi county**: Hsiting, K. C. Yang 5646 (TNM); Bunkiko, U. Faurie s. n. (TI); Mt. Chiananyun, ca. 1600 m, 29 Mar 2012, T. C. Hsu 5525 (TAIF); Tatungshan, 1700 m, 19 Oct 1998, T. W. Hsu 9244 (TAIE); **Yunlin county**: Shihpishan, 1500 m, 13 Mar 2009, C. T. Chao 476, 478 (TCF); **Tainan city**: Tatungshan, T. C. Huang & S. F. Huang 15995 (TAI); en route from Chietung Villa to Tienyunshan, 1300 m, 1 Nov 1985, C. I Peng 8805 (HAST); **Hualien county**: Hoping logging trail 27–27.5 km, ca. 1200 m, 6 May 2000, S. T. Chiu et al. 6163 (TNM); Hoping logging trail 39.7 km, near the working cabin of Forest Bureau, ca. 2000 m, 7 May 2006, S. T. Chiu et al. 6274 (TNM); Hoping logging track, 26–39 km, 800–1800 m, 22 Apr 2002, S. M. Kuo et al. 705 (NTNU); Tentyo cliff, C. H. Chen 7029 (TNM); Monte Taroko-taizan, 1800–2000 m, 14 Jun 1933, T. Suzuki 9494 (TAI); from Fong-shan Branch to no. 29 compt., Liu et al. 96 (TAI); Hoping, ca. 1300 m, 22 Apr 2002, S. M. Kuo et al. 705 (TAIF); Mt. Chienliyen, 1400 m, 17 Apr 2011, T. C. Hsu 3879 (TAIF); from peak of Chingshuishan to Shakatang forest road, 1500–2000 m, W. P. Leu 1804 (HAST); same loc., 1300 m, 30 Apr 2016, C. T. Chao 4110 (TCF); Lanshan, 1500 m, 1 Apr 1994, Y. C. Sun 220 (TAIE); Mt. Mukwashan forest, Halun station, 2000–2100 m, 23 Sep 1984, C. I Peng 7278 (HAST); **Kaohsiung city**: Mt. Hsiaokuan logging trail, 16 Apr 2009, M. J. Jung 3953 (TAIF); **Pingtung county**: Mt. Lili, 1000 m, 6 May 2012, T. C. Hsu 5656 (TAIF); same loc., 30 Apr 2013, T. C. Hsu 6543 (TAIF); on a hiking path near Wutai, 860 m, 26 Feb 2002, W. C. Leong 3019 (HAST); Wutai to Ali, 1100 m, 7 Dec 2015, C. T. Chao 4019 (TCF);

**Figure 6.**
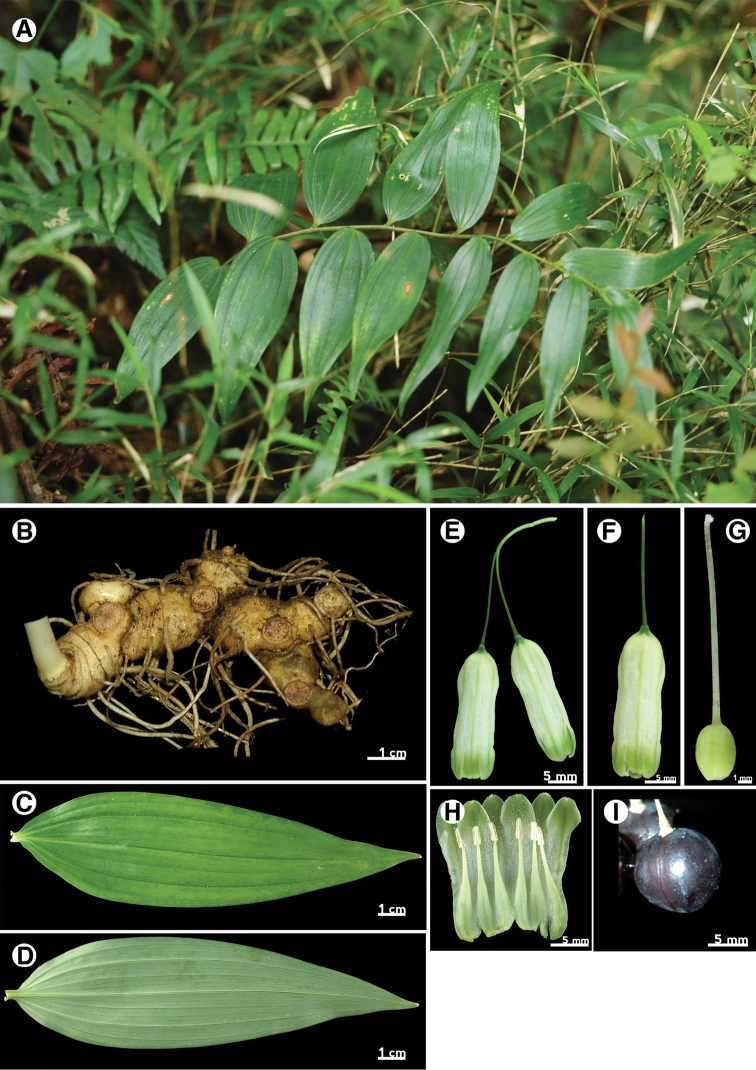
Polygonatumarisanensevar.arisanense. **A** habit **B** rhizome **C** leaf adaxial surface **D** leaf abaxial surface **E** inflorescence **F** flower **G** pistil **H** expanded perianth tube with stamens **I** fruit.

#### 2. Polygonatum arisanense Hayata var. chingshuishanianum

Taxon classificationPlantaeAsparagalesAsparagaceae

(S. S. Ying) C. T. Chao & Y. H. Tseng comb. nov.

urn:lsid:ipni.org:names:77194987-1

Fig. 7 清水山黃精

Polygonatum chingshuishanianum S. S. Ying in Memoirs of the College of Agriculture, National Taiwan University 28(2): 42. 1988. Ying, Fl. Taiwan 5:61, 2000; Boufford et al., Fl. Taiwan 6:112, 2003. Type: Hualien county, Hsiulin township, Chingshuishan, 2300 m, 26 Jun 1988, S. S. Ying s.n. (holotype: NTUF!), **syn. nov.**
Polygonatum altelobatum sensu auct. Wang non Hayata, Icones Plantarum Formosanarum 5:229. 1915.

##### Perennial herbs.

Rhizome tuberous, 1.5–1.8 cm in diam. Stem straight, 10–30 cm long, purplish-green, base covered with scale leaf, caducous. Leaves deciduous, alternate, chartaceous to thinly coriaceous, 3-nerved, lanceolate to oblong-lanceolate, 5.5–7.5 cm long, 1.5–2.5 cm wide, apex obtuse, base obtuse and decurrent to the petiole base, entire, glabrous on both surfaces, petiole ca. 3 mm long, glabrous. Inflorescences solitary or two flowers, peduncle ca. 1.3 cm long. Flowers pendulous, pedicels 5.0–6.5 mm long, glabrous, articulated close to the flower, perianth tube 1–1.5 cm long, ca. 8 mm wide, base flattened, perianth segments 6, arranged into 2 whorled, each 3, triangular, ca. 5 mm long, ca. 5 mm wide, green with dark green strip, apex obtuse, floccose. Stamens 6, base expansion and flattened, inserted at middle of perianth, filaments 5.5–6.5 mm long, anthers oblong-lanceolate, 1.5–2.0 mm long, ca. 1 mm wide. Ovary superior, globose, 4.5–5.5 mm long, 3.5–4.5 mm in diam., glabrous, style filiform, ca. 1 cm long, glabrous, stigma entire, pubescent. Fruits berry, globose, glabrous. 2n=22.

##### Endemic.

Known only from the summit of Mt. Chingshuishan, ca. 2300–2400 m, on exposed limestone slopes (Fig. 4).

##### Specimen examined.

**Hualien county**: Hsiulin township, Mt. Chingshuishan, on the way from the entrance to the mountain peak, 2000–2400 m, 9 May 1997, C. H. Chen & S. D. Shen 2066 (TNM; TNU); Mt. Chingshuishan, near the summit, ca. 2200 m, 2 Jun 2010, C. T. Chao 1424 (TCF); same loc., 17 May 2014, C. T. Chao 3396 (TCF); same loc., 17 May 2015, C. T. Chao 3767 (TCF); same loc., 1 May 2016, C. T. Chao 4128 (TCF); Hsiulin township, 2400 m, 22 Aug 1996, C. H. Chen & C. T. Lu 64 (TNU).

**Figure 7.**
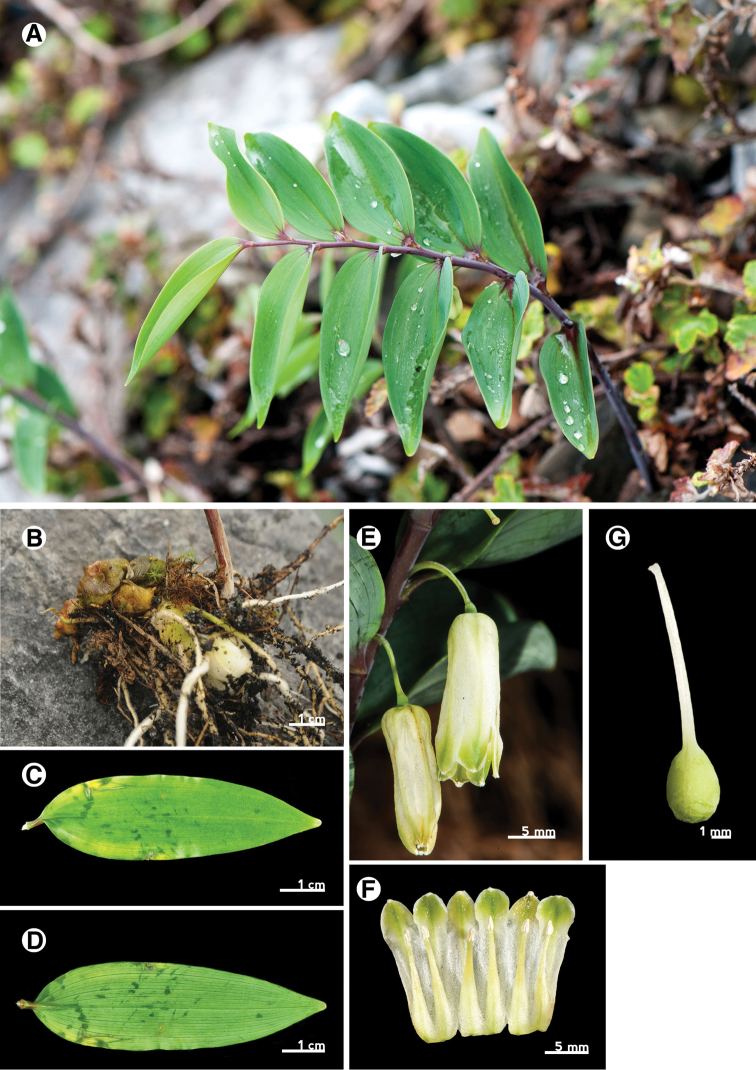
Polygonatumarisanensevar.chingshuishanianum. **A** habit **B** rhizome **C** leaf adaxial surface **D** leaf abaxial surface **E** inflorescence **F** expanded perianth with stamens **G** pistil.

#### 3. Polygonatum arisanense Hayata var. formosanum

Taxon classificationPlantaeAsparagalesAsparagaceae

(Hayata) C. T. Chao & Y. H. Tseng comb. nov.

urn:lsid:ipni.org:names:77194988-1

Fig. 8 大屯黃精

Polygonatum officinale All. var. formosanum Hayata, Icones Plantarum Formosanarum. 9:140. 1920. Type: Taipei City, Taiton (Mt. Tatunshan), ad 1500 metra, U. Faurie 544 (holotype: KYO!).
Polygonatum formosanum (Hayata) Masam. & Simada, Short Flora of Formosa 271. 1936.
Polygonatum daitonense T. S. Liu & S. S. Ying *nom. nud.*

##### Perennial herbs.

Rhizome tuberous. Stem straight to arching, 30–80 cm long, green or purplish, glabrous, covered with scale leaf at the base, caducous. Leaves deciduous, alternate, thick chartaceous to coriaceous, ovate to lanceolate ovate 3-multiple nerved, apex attenuate, base obtuse, 8.0–10.5 cm long, 3.5–5.5 cm wide, sessile or short-petioled, ca. 3 mm long, often purplish. Inflorescences axillary, solitary to umbels with 3–5 flowers, peduncle subequal to pedicels, 1.5–2.0 cm long, articulated close to the flower, bracteoles very minutely, caducous. Flowers pendulous, perianth tube, 2.5–3.5 cm long, 0.5–1.0 cm wide, slight flattened at base, white with green strips, perianth segments 6, arranged into 2 whorls, each 3, 5.5–8.0 mm long, 5.5–8.0 mm wide, green with dark green strip, ovate to triangle, apex obtuse, floccose. Stamens 6, filaments slender, base expansion and inflated, papillose or short cottony, inserted at middle of perianth tube, ca. 5 mm long, anthers 1.5–2.0 mm long, 2-loculed, introrse and longitudinally dehiscent. Ovary superior, 3-loculed, ovate to oblong, covered with white powder, 3.5–4.0 mm long, ca. 3 mm in diam., style filiform, ca. 1.5 cm long, stigma entire, pubescent. Fruit berry, purplish black. 2n=22.

##### Endemic.

Only know from Yangmingshan national park, northern Taiwan (Fig. 5).

##### Specimen examined.

**Taipei city**: Mt. Tatunshan, 26 Apr 1931, S. Sasaki 19 (TAI); same loc., 10 May 1936, H. Simada 1292 (TAI); same loc., 10 Apr 1981, Y. F. Chen 2025 (TAI); same loc., 8 May 2010, C. T. Chao 1416 (TCF); same loc., 1 May 2016, C. T. Chao 4105 (TCF); Tsaoshan to Chutzihu, 1931, Tanaka 1250 (TAI); Shiaokuangyingshan, 11 Apr 1937, H. Simada 1287 (TAI); same loc., 22 Apr 1937, H. Simada 1289 (TAI); Mt. Chihsingshan, 30 Jun 1937, H. Simada 1369 (TAI); same loc., 1 Nov 1968, M. Mizushima et C. C. Hsu 5082 (TAI); same loc., 14 Apr 1983, K. C. Yang 1367 (TAI); same loc., 25 Apr 1985, T. C. Wan & K. C. Yang 2004 (TAI); Chunghsing Farm. 4 Apr 1988, T. C. Huang 13452 (TAI); Chutzuhu, 500 m, 24 Mar 1972, J. W. Tsai 88 (TAIF); Hsiantienhu, 800 m, 16 Sep 1985, S. Y. Lu 16890 (TAIF); Tsaikungkengshan, 850 m, 19 Aug 2001, P. F. Lu 1123 (TAIF); same loc., 26 Oct 2002, S. W. Chung 5943 (TAIF); same loc., 1 Apr 2006, P. F. Lu 11560 (HAST); same loc., 850 m, 14 Jul 1996, S. C. Wu et al. 877 (TNM, TNU); Yangmingshan, 700–800 m, 4 Apr 2008, P. F. Lu 15623 (HAST); Chungchengshan to Tatunnanfeng, 600–900 m, 3 Apr 1998, C. M. Wang 3005 (HAST, TNM); Palaka highway, close to Hsiaoyukeng, 700–800 m, 24 Feb 1997, S. M. Kuo & H. M. Shih 79 (TNU); on the top of Mt. Chihsing, 900–1000 m, 28 Apr 1997, S. M. Kuo & Y. C. Chen 81 (TNU); the path to Tsaikungkengshan, 700–800 m, 30 Mar 2007, Y. C. Huang 546 (TNU);

**Figure 8.**
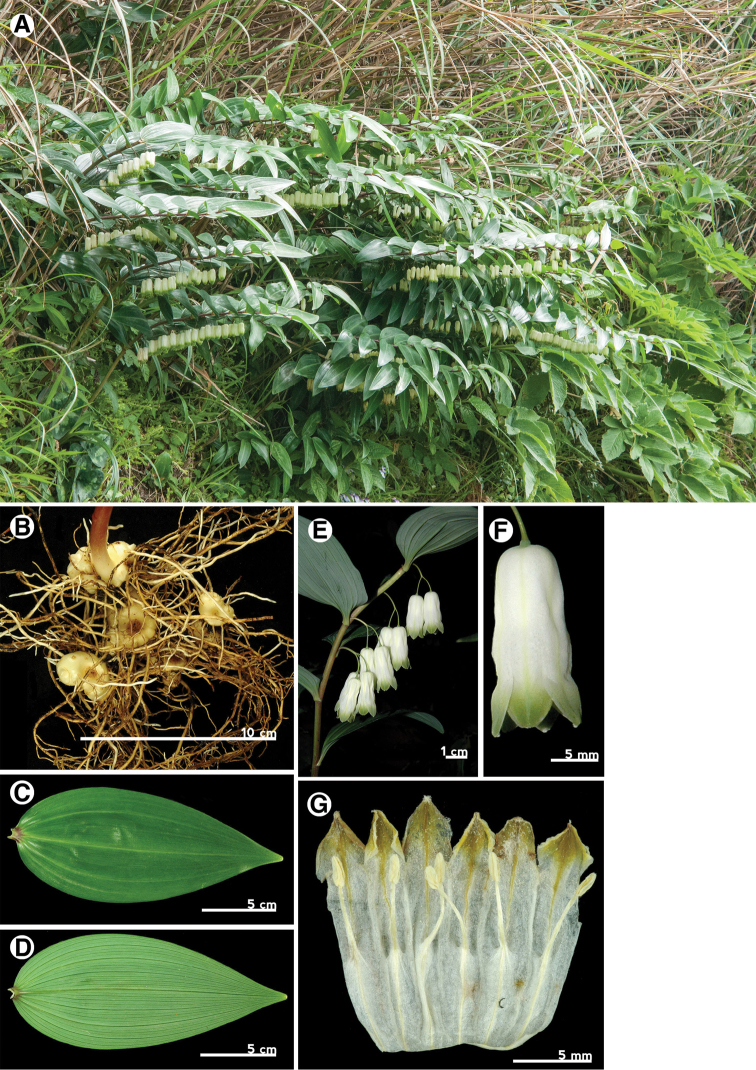
Polygonatumarisanensevar.formosanum. **A** habit **B** rhizome **C** leaf adaxial surface **D** leaf abaxial surface **E** inflorescences **F** flower **G** expanded perianth with stamens.

## Supplementary Material

XML Treatment for Polygonatum arisanense Hayata var. arisanense

XML Treatment for Polygonatum arisanense Hayata var. chingshuishanianum

XML Treatment for Polygonatum arisanense Hayata var. formosanum
